# Propagation graph estimation from individuals’ time series of observed states

**DOI:** 10.1038/s41598-022-10031-3

**Published:** 2022-04-12

**Authors:** Tatsuya Hayashi, Atsuyoshi Nakamura

**Affiliations:** Graduate School of Information Science and Technology, Hokkaido University, Sapporo, 060-0814 Japan

**Keywords:** Computer science, Information technology, Scientific data

## Abstract

Various things propagate through the medium of individuals. Some individuals follow the others and take the states similar to their states a small number of time steps later. In this paper, we study the problem of estimating the state propagation order of individuals from the real-valued state sequences of all the individuals.We propose a method of constructing a state propagation graph from individuals’ time series of observed states. The propagation order estimated by our proposed method is demonstrated to be significantly more accurate than that by a baseline method (optimal constant delay model) for our synthetic datasets, and also to be consistent with visually recognizable propagation orders for the dataset of Japanese stock price time series and biological cell firing state sequences.

## Introduction

Sometimes, it is very important to analyze how things such as vibration, heat, cell firing, information, virus and etc, propagated. The objectives of such analyses are diverse from identification of the sources and the propagation routes to learning a propagation model for prediction. Physical propagation such as vibration and heat follows physical law. However, biological propagation such as cell firing has more ambiguous propagation rules, and propagation through the medium of human beings such as information and virus propagation is more complex.

The state propagation from one individual to another individual can be seen as a simple causal relationship between them. Granger causality^1^ and transfer entropy^2^ are well-known methods for investigating the causal relationship between time series, and their extensions and applications have been still energetically investigated^3–5^. In these methods, a parameterized stationary model is assumed and long time series are needed for its parameter estimation. Contrary to the fact that these methods can deal with various kinds of influence, the state propagation treats only the influence of taking similar states with some delay. By virtue of this simplicity, propagation relation estimation does not need such long time series.

In this study, we propose an alignment-based method of estimating state propagation relationship between a pair of individuals from their time series of observed states. There already has existed an extended Granger causality method into which a kind of alignment called dynamic time warping (DTW) is incorporated to deal with the arbitrary-time-lag influence between time series^6^. Different from this Granger causality extension, we estimate time delays of the propagations and use them to estimate direct and indirect propagations. Time delay estimation among signals^7,8^ has been studied well in the context of source localization, however, only constant time delays are dealt with there. We treat variable time delays and estimate time delay sum.

From the estimated state propagation relationships between all the pairs of individuals, we construct an estimated state propagation graph whose edges are composed of the estimated direct propagations only. As for propagations through networks, various information or influence propagations have been studied: word-of-mouth marketing^9–12^, epidemics^13–15^, innovation diffusion^16,17^ and so on. In most of these studies, networks are assumed to be given and not needed to be estimated though there are studies on propagation probability estimation through edges in a given network^18–22^. Studies on propagation through social networks are popular^23–25^, but in most social networks, relation between users are visible and not needed to be estimated. Recently, methods to reconstruct a complex network from binary time series have been developed^26,27^, but those methods require the sufficient length of binary time series because they use the maximum-likelihood estimation of the probabilities associated with presence or absence of links.

In our proposed method, for each pair of individuals (*i*, *j*), we calculate the time delay sum of individual *j*’s states from individual *i*’s matched states averaged over all the minimum cost alignments between their state time series. Then, propagation direction between *i* and *j* is estimated as $i\rightarrow j$ if such averaged time delay sum is positive, and as $j\rightarrow i$ if it is negative. From individual pairs (*i*, *j*) with non-zero average time delay sum, we construct an estimated propagation graph whose vertices are individuals and whose edges are estimated direct propagation. In the construction, in order to exclude indirect propagation edges, we greedily remove the edge (*i*, *j*) with the largest average time delay sum if there is an indirect path from *i* to *j* and the delay is at least an estimated upper bound of direct propagation $\theta$, and remove all the edges between vertices in the same estimated layer.

According to our experiments using real-valued and binary-valued time series synthetic datasets generated by stochastic delay models, the edge sets of propagation graphs estimated by our method achieved comparable or higher F-measure and *layer accuracy* than those by a baseline method (optimal constant delay model), where layer accuracy is the accuracy of the estimated number of steps to be taken for propagation from the source individuals to each individual. In order to demonstrate practical usefulness of our method, we applied our method to propagation analyses of stock price and biological cell firing. For both datasets, the propagation order estimated by our proposed method is shown to be consistent with visually recognizable propagation order. The propagation delay is not stable for stock price propagation, but which stocks tended to follow which stocks in a given period is interesting information and automatic visualization may be useful to investors. Our method is considered to be useful for analyses of such unstable propagation.

## Methods

### Problem setting

Let *I* denote a set of individuals $\{1,\dots ,N\}$. We let [*n*] denote $\{1,\dots ,n\}$ for any positive integer *n*, so $I=[N]$. At each time step $t=1,\dots ,T$, each individual $i\in I$ takes state $s_i[t]\in Y$, where $Y={\mathbb {R}}$ or $\{0,1\}$. Let ${\mathbf {s}}_i$ denote the state time series of length *T* whose *t*th value is $s_i[t]$, that is, ${\mathbf {s}}_i=s_i[1]\cdots s_i[T]$. We consider the following state propagation between individuals. Assume that there exist source individuals and the states propagate from individuals to individuals at each time. As for state propagation, we assume the following.

#### Assumption 1

Each individual *i* but the source individuals, follows some other individuals *j*, and the follower *i* takes state $s_i[t]$ similar to state $s_j[t-\Delta _{i,j}[t]]$ with small time step delay $\Delta _{i,j}[t]$ at each time step *t*.

Note that, in real applications, ${\mathbf {s}}_i$ is composed of periodically sampled values and the number and interval of sampling are very important issues to detect the direction of propagation. In this paper, we do not argue those issues and assume that appropriate number and interval of sampling are taken to construct the state time series.

The state propagation can be represented by a *state propagation graph* *G*(*V*, *E*) with vertex set $V=I$ and directed edge set $E=V\times V$, in which directed edge $(i,j)\in E$ exists if and only if individual *j* directly follows *i*. The problem we try to solve in this paper is formalized as follows.

#### Problem 1

Given a set $\{{\mathbf {s}}_1,\dots ,{\mathbf {s}}_N\}$ of the length-*T* state time series of individuals in $I=[N]$, estimate the state propagation graph with vertex set *I* under Assumption 1.

Note that, considering that *V* is fixed to *I*, a solution of the above problem is estimation ${\hat{E}}$ of the directed edge set *E*.

### Alignment-based direction estimation

Let ${\mathbf {s}}_i$ and ${\mathbf {s}}_j$ be the state time series of individuals *i* and *j*. From ${\mathbf {s}}_i$ and ${\mathbf {s}}_j$, we estimate in which direction $i\rightarrow j$ or $j\rightarrow i$ the states propagated. As an estimation method, we propose a method based on the sum of delay times at matched positions in the minimum cost alignments between ${\mathbf {s}}_i$ and ${\mathbf {s}}_j$. An alignment of two time series ${\mathbf {s}}_i$ and ${\mathbf {s}}_j$ is a pair of two same length sequences ${\mathbf {s}}'_i$ and ${\mathbf {s}}'_j$ which are made from ${\mathbf {s}}_i$ and ${\mathbf {s}}_j$, respectively, by inserting some values at some positions in ${\mathbf {s}}_i$ and ${\mathbf {s}}_j$ so as to take similar values at the same positions. As an inserted value, two types of values are considered: a gap $\text{\textvisiblespace}$ in gap-based alignment and the same value as the previous-position’s value in DTW(dynamic time warping)-based alignment. For example, one of gap-based alignments between two binary-state time series ${\mathbf {s}}_i=001000100$ and ${\mathbf {s}}_j=000100010$ is

1 $$\begin{aligned} \begin{array}{lllllllllll} \text {position in } {\mathbf {s}}_i &{} &{}1&{}2&{}3&{}4&{}5&{}6&{}7&{}8&{}9\\ {\mathbf {s}}'_i &{} \textvisiblespace &{}0&{}0&{}1&{}0&{}0&{}0&{}1&{}0&{}0\\ {\mathbf {s}}'_j &{} 0&{}0&{}0&{}1&{}0&{}0&{}0&{}1&{}\textvisiblespace &{}0\\ \text {position in } {\mathbf {s}}_j &{} 1&{}2&{}3&{}4&{}5&{}6&{}7&{}8&{}&{}9\\ \text {matched position} &{} &{}*&{}*&{}*&{}*&{}*&{}*&{}*&{}&{}*\end{array} \end{aligned}$$

and one of DTW-based alignments between two real-state time series ${\mathbf {s}}_i=2210022310$ and ${\mathbf {s}}_j=1221022231$ is

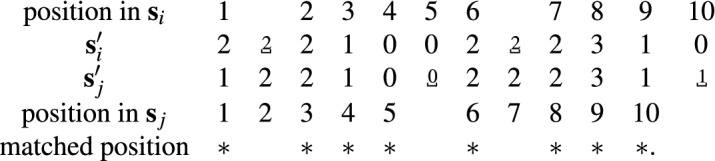



There are various alignments between a pair of time series but only the minimum cost alignments are considered for a given cost function $w: (Y\cup \{\textvisiblespace \})^2\rightarrow {\mathbb {R}}$, where the cost of the alignment $({\mathbf {s}}'_i, {\mathbf {s}}'_j)$ is defined as $\sum _{t=1}^{T'}w(s'_i[t],s'_j[t])$ for the length $T'$ of the aligned sequences ${\mathbf {s}}'_i$ and ${\mathbf {s}}'_j$. As for a cost function, we use the absolute difference $w(x,y)=|x-y|$ in a DTW-based alignment. In a gap-based alignment, we use a problem dependent cost function. For example, in the case that $Y=\{0,1\}$ and each 1-state in one sequence is strongly preferred to be aligned to 1-state in the other sequence by shifting positions unless their position difference is large ($2\times \text {(position difference)}>\alpha$) or the number of 1-states is different, the following cost function seems to be appropriate:

2 $$\begin{aligned} w(x,y) = {\left\{ \begin{array}{ll} 0 &{} ((x,y)=(0,0),(1,1))\\ 1 &{} ((x,y)=(0,\textvisiblespace ),(\textvisiblespace ,0))\\ \alpha &{} ((x,y)=(0,1),(1,0))\\ \infty &{} ((x,y)=(1,\textvisiblespace ),(\textvisiblespace ,1)(\textvisiblespace ,\textvisiblespace )). \end{array}\right. } \end{aligned}$$

For the cost function (Eq. 2) with $\alpha =3$, the cost of the alignment (Eq. 1) is 2, which is a minimum cost alignment. There are 6 minimum cost alignments between the time series ${\mathbf {s}}_i=001000100$ and ${\mathbf {s}}_j=000100010$ for the cost function. Let $M({\mathbf {s}}'_i,{\mathbf {s}}'_j)$ denote the set of matched position pairs in the alignment $({\mathbf {s}}'_i,{\mathbf {s}}'_j)$. For example, $M({\mathbf {s}}'_i,{\mathbf {s}}'_j)$ for the alignment (Eq. 1) is $\{(1,2),(2,3),(3,4),(4,5),(5,6),(6,7),$ $(7,8),(9,9)\}$. Define the *time delay* of a matched position pair $(t'_i,t'_j)$ by $t'_j-t'_i$, and consider the *time delay sum* of ${\mathbf {s}}'_j$ from ${\mathbf {s}}'_i$ calculated by $\sum _{(t'_i,t'_j)\in M({\mathbf {s}}'_i,{\mathbf {s}}'_j)}(t'_j-t'_i)$. For example, the time delay sum of ${\mathbf {s}}'_j$ from ${\mathbf {s}}'_i$ for the alignment (Eq. 1) is $1+1+1+1+1+1+1+0=7$. The time delay sums for the other 5 minimum cost alignments are 5, 6, 6, 7, 8, so the time delay sum averaged over all the 6 minimum cost alignments is 6.5.

Using the average time delay sum of the minimum cost alignments, we estimate the direction of state propagation between individuals *i* and *j* by the following rule (E).

(E) The propagation direction is estimated as $i\rightarrow j$ if the time delay sum of ${\mathbf {s}}'_j$ from ${\mathbf {s}}'_i$ averaged over the minimum cost alignments $({\mathbf {s}}'_i,{\mathbf {s}}'_j)$ between ${\mathbf {s}}_i$ and ${\mathbf {s}}_j$ is positive, and $j\rightarrow i$ if that is negative.

### Edge set estimation

By rule (E), directions are decided for all the individual pairs but those with zero average time delay sum. If we let the estimated edge set ${\hat{E}}$ be the set of all $(i,j)\in I\times I$ with non-zero average time delay sum, the following two issues arise:

1. ${\hat{E}}$ contains many edges with small average time delay sum, which connects pairs of synchronized individuals.
2. ${\hat{E}}$ contains (*i*, *j*) for which individual *i*’s state not directly but indirectly affects individual *j*’s state through the medium of some other individual *k*.

As a countermeasure for P2, that is, in order to delete indirectly affecting edges, we define a candidate edge as an edge with average time delay sum larger than threshold $\theta$ and sort all the candidate edges by average time delay sum in descending order and greedily delete edge (*i*, *j*) one by one for which an indirect path from *i* to *j* exists. Threshold $\theta$ should be set to the estimated maximum average time delay sum of *directly* affecting edges. In the distribution over average time delay sum between all the individual pairs, average time delay sum between directly affecting pairs is considered to form the highest peak with high probability. So, we set $\theta$ to the first valley position larger than the highest peak position in the distribution of the average time delay sum estimated by kernel density estimation.

For P1, we try to partition *V* into layers by classifying the synchronized individuals to the same layer, and then delete all the edges between vertices in the same layer. For a given graph *G*(*V*, *E*), define the 0-layer set $V^E_0$ as the set of vertices with indegree 0. If there is no vertex with indegree 0, define $V^E_0$ as the set of vertices for which the maximum average time delay sum among all the incoming edges is the smallest among those for all the vertices. Define the *i*-layer set $V^E_i$ recursively as the set of vertices that do not belong to the *j*-layer set $V^E_j$ for any $j=0,1,\ldots ,i-1$ but have an incoming edge from some vertex in the $(i-1)$-layer set $V^E_{i-1}$.

Given a graph $G(V,{\hat{E}})$ with $V=I$ and the set ${\hat{E}}$ of directed edges *e* whose direction is estimated by its average time delay sum ${\text {AD}}(e)$, and threshold $\theta$, the whole process of edge set estimation is described as follows.

1. $e_1,\ldots ,e_m\leftarrow$ sorted list of edges $e\in {\hat{E}}$ with ${\text {AD}}(e)>\theta$ in descending order of ${\text {AD}}(e)$.
2. For $e=e_1,\dots ,e_m$, remove the edges $e=(i,j)\in {\hat{E}}$ if there exists an indirect path from *i* to *j*.
3. Set $V_0^{{\hat{E}}}$ to the set of vertices in *V* whose indegree is 0.
4. Set *i* to 1. Repeat the followings until $V\setminus \bigcup _{j=0}^{i-1}V_j^{{\hat{E}}}=\emptyset$: set $V_i^{{\hat{E}}}$ to the set of vertices in $V\setminus \bigcup _{j=0}^{i-1}V_j^{{\hat{E}}}$ that has an incoming edge from a vertex in $V_{i-1}^{{\hat{E}}}$, and then increase *i* by 1.
5. Remove all the edges $(i,j) \in {\hat{E}}$ whose end points *i*, *j* belong to the same layer $V_k^{{\hat{E}}}$ for some $k\in [N]$.

### Example

**Figure 1:**
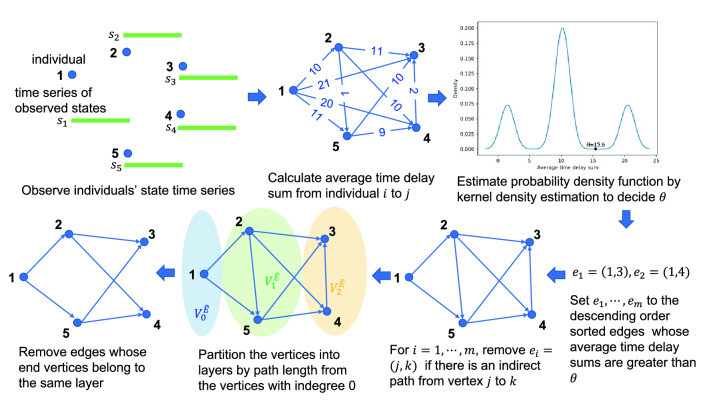
Summary diagram of our method with a toy example.

Figure 1 is the summary diagram of our method with a toy example. In the example, state time series $s_1,\dots s_5$ for five individuals $1,\dots ,5$ are assumed to be observed and the average time delay sum of every pair is calculated. (The number written on each edge indicates its average time delay sum.) Threshold $\theta$ is set to 15.6 because it is the first valley position larger than the highest peak position around 10 in the distribution of the average time delay sum estimated from the set of the average time delay sums $\{1,2,9,10,10,10,11,11,20,21\}$ using kernel density estimation. In this case, the average time delay sum 20 and 21 of edges (1, 3) and (1, 4), respectively, are more than $\theta$, so in descending order of average time delay sum, first, edge (1, 3) is checked if there exists an indirect path from vertex 1 to vertex 3 and removed because there exists, then, edge (1, 4) is checked similarly and removed. Finally, the five vertices are divided into three layers by their path lengths from vertex 1, which is the vertex with indegree 0, and the estimated edge set ${\hat{E}}$ is made by removing all the edges between the same layer’s vertices. In the last procedure, edges (2, 5) and (4, 3) are removed in our example.

## Numerical simulations and application to real-world datasets

In this section, we experimentally show effectiveness of our method using synthetic and real world datasets. The gap-based cost *w* defined by Eq. (2) with $\alpha =3$ is used by the proposed method using gap-based cost in all the experiments for binary state propagation. Generating graphs and plots in our experiments was executed in Mathematica^28^.

### Experiments using synthetic datasets

First, we evaluate how accurate the estimated edge set ${\hat{E}}$ by the proposed method is for the real-valued and binary state sequence dataset generated from a delay model with a given ground truth propagation graph *G*(*V*, *E*).

#### Ground truth graphs and datasets

**Figure 2:**
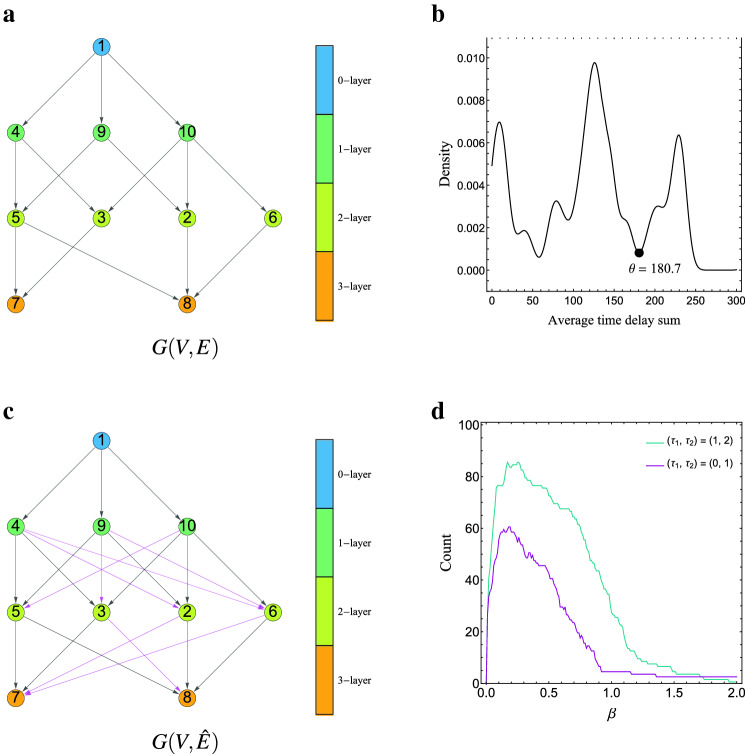
(**a**) Ground truth graph *G*(*V*, *E*) in the experiment of real-valued state propagation using synthetic datasets. (**b**) The probability density of average time delay sum estimated by kernel density estimation for one of the synthetic datasets. The black circle ($\theta = 180.7$) indicates a threshold value adopted by our method. (**c**) The estimated graph $G(V,{\hat{E}})$ from one of the datasets by the proposed method. In $G(V,{\hat{E}})$, black and magenta arrows are edges in *E* and ${\hat{E}}\setminus E$, respectively. Each vertex’s color indicates its belonging layer. (**d**) The number of datasets in which parameter $\beta$ of the bandwidth achieves the minimum MLD.

**[Real-valued State Propagation]** We generate the dataset using ground truth propagation graph *G*(*V*, *E*) shown in Fig. 2a. The length-100 time series $s_i[1]\cdots s_i[100]$ for each vertex (individual) $i=1,\dots ,10$ are generated by the following steps. Note that $\text {in}(i)$ denotes the set of vertices from which edges come to vertex *i*.

Step 1 Generate an i.i.d. sequence $s_1[1],\dots ,s_1[100]\sim N(0,5^2)$.
Step 2 Generate the sequence ${\mathbf {s}}_i$ as follows in the order of $i=4,9,10,2,3,5,6,7,8$:
  1. $s_i[1],s_i[2]\sim N(0,5^2)$, $\Delta _{j,i}[2]\leftarrow \tau _1$ or $\tau _2$ ($\tau _1, \tau _2 \in {\mathbf {Z}}_{\ge 0}$) randomly for $j\in \text {in}(i)$.
  2. For $t=3,4,\dots ,100$ and $j\in \text {in}(i)$, generate $s_i[t]$ as
    $$\begin{aligned} \Delta _{j,i}[t]\leftarrow&{\left\{ \begin{array}{ll}\Delta _{j,i}[t-1] &{} \text { with prob. } 3/4\\ \tau _1 + \tau _2 - \Delta _{j,i}[t-1] &{} \text { with prob. } 1/4\end{array}\right. }\\ \varepsilon \leftarrow&\text {random value generated according to } N(0,1)\\ s_i[t]\leftarrow&\left( \sum _{j\in \text {in}(i)}s_j[t-\Delta _{j,i}[t]]\right) /|\text {in}(i)|+\varepsilon . \end{aligned}$$

We generated 100 datasets using this procedure in our experiment for each $(\tau _1,\tau _2)=(1,2),(0,1)$. Note that $\tau _1$ and $\tau _2$ are two possible time delays and $\Delta _{j,i}[t]\in \{\tau _1,\tau _2\}$ holds for all $i=2,\dots ,10$, $j\in \text {in}(i)$ and $t=2,\dots ,100$.

**[Binary state propagation]** The dataset is generated by propagation model in which individuals are located in 2-dimensional real space and state-1 of individual *j* is propagated from individuals *i* within some distance, then the ground truth graph *G*(*V*, *E*) is generated from the dataset and individuals’ location information. Note that the proposed method estimates *E* without individuals’ location information. Given a parameter $0<p\le 1$ of the state-1 propagation probability, the length-200 time series $s_i[1]\cdots s_i[200]$ for each vertex (individual) $i=1,\dots ,50$ is generated as follows.

Step 1 For $i=1,\dots ,50$, the location $r_i$ of individual *i* is randomly selected according to uniform distribution over $[0,M]^2$.
Step 2 For $t=1,\dots ,200$, set $s_1[t]=1$ if $t\%10 = 1$ and set $s_1[t]=0$ otherwise, where $\%$ is modulus operator.
Step 3 For $i=2,\dots ,50$ and $t=1,\dots ,200$, set $s_i[t]=1$ with probability *p* if the following two conditions
  1. $\exists j$ s.t. $\Vert r_j-r_i\Vert \le 35$, $s_j[t-1]=1$ (there is an individual within distance 35 that takes state 1 at just one step before) and
  2. $s_i[t-k]=0$ for all $k=1,2,\dots ,\min \{5,t-1\}$ (state-1 interval of each individual is at least 5),
  are satisfied and set $s_i[t]=0$ otherwise.

From the dataset $\{{\mathbf {s}}_1,\dots ,{\mathbf {s}}_{50}\}$ generated above and location information $\{r_1,\dots ,r_{50}\}$, edge set *E* of the ground truth propagation graph *G*(*V*, *E*) is created as follows. Let *n*(*i*, *j*) denote the number of individual *j*’s state 1 caused by individual *i*’s state 1, that is,

$$\begin{aligned} n(i,j)=|\{t \in \{1,\dots ,200\}\mid s_i[t-1]=1, s_j[t]=1, \Vert r_j-r_i\Vert <35 \}|, \end{aligned}$$

where $|\cdot |$ denotes the number of elements in set ‘$\cdot$’. Then, *E* is defined as

$$\begin{aligned} E=\{(i,j)\in V\times V\mid n(i,j)>n(j,i)\}. \end{aligned}$$

**Figure 3:**
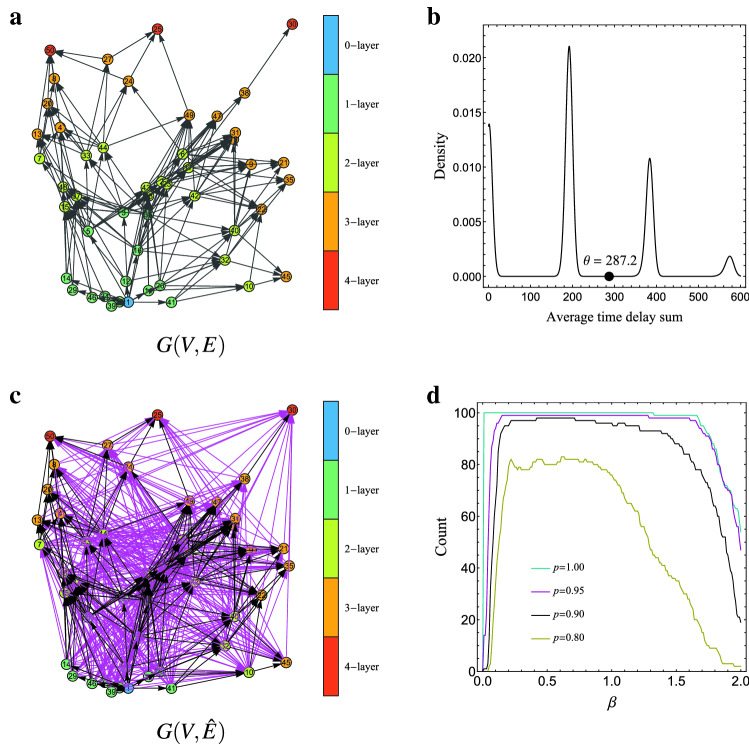
(**a**) The ground truth graph *G*(*V*, *E*) in the experiment of binary state propagation using one of the synthetic datasets with $p=0.95$. (**b**) The probability density of average time delay sum estimated by kernel density estimation for the dataset. The black circle ($\theta = 287.2$) indicates a threshold value adopted by our method. (**c**) The estimated graph $G(V,{\hat{E}})$ by the proposed method for the dataset. Each individual *i* is located at $r_i$. In $G(V,{\hat{E}})$, black arrows are edges in *E* and magenta arrows are edges in ${\hat{E}}\setminus E$. Note that ${\hat{E}}$ includes *E* (recall 1.0) for this dataset. Each individual’s color indicates its belonging layer: blue, green, yellow, orange and red individuals belong to $V^{{\hat{E}}}_0$, $V^{{\hat{E}}}_1$, $V^{{\hat{E}}}_2$, $V^{{\hat{E}}}_3$, and $V^{{\hat{E}}}_4$, respectively. (**d**) The number of datasets in which parameter $\beta$ of the bandwidth achieves the minimum MLD.

A ground truth graph *G*(*G*, *E*) for one dataset with $p=0.95$ is shown in Fig. 3a.

In the experiment, we generate 100 datasets and corresponding ground truth graphs for each $p=1.00, 0.95, 0.90, 0.80, 0.70,$ 0.60, 0.50.

#### Evaluation measures

As a direct evaluation measure of delay estimations, we define *mean absolute error of average time delay (MAEATD)* as follows. For $(i,j)\in E$, define $D_{i,j}$ as $D_{i,j}=\sum _{t=a}^{T}\Delta _{i,j}[t]$ and let ${\hat{D}}_{i,j}$ denote its estimation, where *a* is the maximum possible time delay in the ground truth model. Then, MAEATD for estimations is defined as $\text {MAEATD}=\frac{1}{|E|(T-a)}\sum _{(i,j)\in E}|{\hat{D}}_{i,j}-D_{i,j}|$.

Using directed edge set *E* of the ground truth propagation graph, we evaluate an estimated directed edge set ${\hat{E}}$ in terms of *precision (Prec)*, *recall (Rec)* and *F*-*measure (FM)* defined as

$$\begin{aligned} \text {Prec}= \frac{|E \cap {\hat{E}}|}{|{\hat{E}}|},\ \text {Rec} = \frac{|E \cap {\hat{E}}|}{|E|} \text { and } \text {FM} = \frac{2\,\,\text {Prec} \cdot \text {Rec}}{\text {Prec} +\text {Rec}}. \end{aligned}$$

Note that our method cannot rank the edges, so evaluation using precision-recall or ROC curve is difficult. How to balance precision and recall depending on applications is one of our future research issues.

In our setting, time series of vertices in the same layer are similar to each other even if their incoming edges are different. In that sense, it is impossible to correctly guess incoming edges, that is, from which vertices in the previous layer the states were propagated directly. Thus, we also evaluate ${\hat{E}}$ in terms of looser measures. We can also consider layer partition $V_0^{{\hat{E}}}, V_1^{{\hat{E}}},\cdots$ for $G(V,{\hat{E}})$ like layer partition $V_0^E, V_1^E,\cdots$ that is defined in the section titled “Edge Set Estimation” for the ground truth propagation graph *G*(*V*, *E*). Then, we define *layer accuracy (LA)* and *Mean layer difference (MLD)* of ${\hat{E}}$ as

$$\begin{aligned} \text {LA}=\frac{\sum _{i=0}^N|V^E_i\cap V^{{\hat{E}}}_i|}{|V|} \text { and } \text {MLD}=\frac{\sum _{i=1}^N|\ell ^E(i)-\ell ^{{\hat{E}}}(i)|}{N}, \end{aligned}$$

where $\ell ^E(i)$ denote the individual *i*’s belonging layer in *G*(*V*, *E*), that is, $\ell ^E(i)=j {\mathop {\Leftrightarrow }\limits ^{\text {def}}} i\in V^E_j$.

As a baseline method, we consider a method outputs Minimum Mean Squared Error (MMSE) constant time delay ${\hat{D}}_{i,j}$ of individual *j*’s states from individual *i*’s states^29^, which is defined as

$$\begin{aligned} {\hat{D}}_{i,j}=\mathop {arg~min}\limits _{-T/2<\Delta \le T/2}\sum _{t=1}^{T}(s_j[t]-s_i[(t+(T-1)-\Delta )\%T+1])^2, \end{aligned}$$

where $\%$ is modulus operator. If there are multiple candidates for ${\hat{D}}_{i,j}$, we adopt ${\hat{D}}_{i,j}$ with the smallest absolute value. Using ${\hat{D}}_{i,j}$, propagation direction is estimated as $i\rightarrow j$ if ${\hat{D}}_{i,j}>0$ and $j\rightarrow i$ if ${\hat{D}}_{i,j}<0$. We construct estimated edge set ${\hat{E}}$ of the baseline method by applying the procedure proposed in the section titled “Edge Set Estimation” using ${\hat{D}}_{i,j}$ instead of the average time delay sum of $s_j$ from $s_i$.

#### Parameters of kernel density estimators

In all the experiments, we use Gaussian kernel in the kernel density estimation. The results of all the simulations were almost the same for other kernels: Biweight, Cosine, Epanechnikov and Triangular. As for the bandwidth *h*, we use the following rule of thumb:

$$\begin{aligned} h = \beta \min \left( {\hat{\sigma }}, \frac{Q_3-Q_1}{1.34}\right) n^{-\frac{1}{5}}, \end{aligned}$$

where $\beta$ is a positive constant, ${\hat{\sigma }}$ is the standard deviation of the samples, $Q_1$ and $Q_3$ are the lower and upper quartiles, respectively, and *n* is the sample size. Constant $\beta$ is set to 0.9 in Silverman’s rule of thumb^30^. In the experiments using synthetic datasets, $\beta$ is set to the value with the minimum MLD that is found by a grid search in $\{0.01, 0.02, 0.03, \dots , 1.99, 2.00 \}$.

#### Results

**[Real-valued state propagation]** Performance comparison with the baseline method by the evaluation measures is shown in Table 1.

**Table 1:** Estimation performance of the baseline method and our proposed method using warping-based cost averaged over 100 datasets. We used Gaussian kernel with a bandwidth with the best $\beta$.

$(\tau _1, \tau _2)$	Method	MAEATD	Prec	Rec	FM	LA	MLD
(1, 2)	Baseline	1.487 ($\pm 0.004$)	0.629 ($\pm 0.015$)	0.889 ($\pm 0.037$)	0.733 ($\pm 0.023$)	0.929 ($\pm 0.033$)	0.081 ($\pm 0.040$)
(1, 2)	Proposed	0.289 ($\pm 0.013$)	0.624 ($\pm 0.011$)	0.836 ($\pm 0.029$)	0.711 ($\pm 0.017$)	0.941 ($\pm 0.015$)	0.060 ($\pm 0.016$)
(0, 1)	Baseline	0.498 ($\pm 0.004$)	0.394 ($\pm 0.026$)	0.357 ($\pm 0.026$)	0.371 ($\pm 0.025$)	0.360 ($\pm 0.043$)	0.719 ($\pm 0.053$)
(0, 1)	Proposed	0.191 ($\pm 0.012$)	0.515 ($\pm 0.024$)	0.620 ($\pm 0.035$)	0.560 ($\pm 0.028$)	0.796 ($\pm 0.034$)	0.225 ($\pm 0.043$)

Note that the values in the table are averaged over 100 datasets and the parenthesized values are their $95\%$ confidence intervals. Our method significantly outperforms the baseline method in all the measures for $(\tau _1,\tau _2)=(0,1)$ and have comparable performance to it for $(\tau _1,\tau _2)=(1,2)$. The reason for performance degrade of the baseline method in the case with $(\tau _1,\tau _2)=(0,1)$ is guessed to be its coarse estimation; it can distinguish one-layer (direct) and two-layer (indirect) propagation differences for $(\tau _1,\tau _2)=(1,2)$ because their expected average delay times are 1.5 and 3 whose nearest integer sets are $\{1,2\}$ and $\{3\}$, respectively, so no intersection exists between them, but for $(\tau _1,\tau _2)=(0,1)$, it cannot distinguish their differences because their expected average delay times are 0.5 and 1 whose nearest integer sets are $\{0,1\}$ and $\{1\}$, respectively, so 1 is a common value. The estimation of our proposed method is fine enough for distinguishing such differences.

The estimated propagation graph $G(V, {\hat{E}})$ by the proposed method for one of the synthetic datasets is shown in Fig. 2c. Parameter $\theta$ is set to 180.7 from the estimated distribution (Fig. 2b). For this dataset, there are some falsely detected edges but all the edges in *E* are correctly detected and all the falsely detected edges keep the correct layer structure. (Fig. 2c). The frequencies of the best grid values $\beta$ for the bandwidth of kernel density estimation are shown in Fig. 2d, which says that $\beta =0.15\sim 0.30$ are appropriate for these datasets.

**[Binary state propagation]**

Performance comparison with the baseline method by our evaluation measures is shown in Table 2. The proposed method outperformed the baseline method in all the measures except precision and for all the *p* values except 0.5. Precisions of both the methods are low compared to their recalls, that is due to correct edge (directly affecting edge) definition: location information is used to define the ground truth graph edges but such information is not used in this experimental setting. Our method successfully estimates each individual’s belonging layer with high LA and low MLD when *p* is around 1 and keeps LA about 0.8 even for $p=0.6$.

**Table 2:** Estimation performance of the baseline method and our proposed method using gap-based cost averaged over 100 datasets for 7 values of parameter *p*:0.50, 0.60, 0.70, 0.80, 0.90, 0.95, 1.00. We used Gaussian kernel with a bandwidth with the best $\beta .$

*p*	Method	Prec	Rec	FM	LA	MLD
1.00	Baseline	0.296 ($\pm 0.020$)	0.960 ($\pm 0.034$)	0.431 ($\pm 0.015$)	0.940 ($\pm 0.047$)	0.122 ($\pm 0.097$)
1.00	Proposed	0.291 ($\pm 0.009$)	1.000 ($\pm 0.000$)	0.449 ($\pm 0.010$)	1.000 ($\pm 0.000$)	0.000 ($\pm 0.000$)
0.95	Baseline	0.298 ($\pm 0.011$)	0.643 ($\pm 0.023$)	0.404 ($\pm 0.013$)	0.938 ($\pm 0.046$)	0.109 ($\pm 0.083$)
0.95	Proposed	0.315 ($\pm 0.011$)	0.991 ($\pm 0.017$)	0.476 ($\pm 0.013$)	0.990 ($\pm 0.020$)	0.020 ($\pm 0.040$)
0.90	Baseline	0.324 ($\pm 0.013$)	0.427 ($\pm 0.021$)	0.363 ($\pm 0.015$)	0.915 ($\pm 0.047$)	0.141 ($\pm 0.084$)
0.90	Proposed	0.314 ($\pm 0.011$)	0.998 ($\pm 0.002$)	0.475 ($\pm 0.013$)	0.990 ($\pm 0.020$)	0.017 ($\pm 0.033$)
0.80	Baseline	0.347 ($\pm 0.018$)	0.308 ($\pm 0.016$)	0.320 ($\pm 0.015$)	0.862 ($\pm 0.048$)	0.217 ($\pm 0.085$)
0.80	Proposed	0.310 ($\pm 0.012$)	0.966 ($\pm 0.022$)	0.466 ($\pm 0.015$)	0.949 ($\pm 0.040$)	0.084 ($\pm 0.068$)
0.70	Baseline	0.353 ($\pm 0.021$)	0.282 ($\pm 0.017$)	0.300 ($\pm 0.017$)	0.771 ($\pm 0.052$)	0.329 ($\pm 0.083$)
0.70	Proposed	0.306 ($\pm 0.014$)	0.906 ($\pm 0.027$)	0.453 ($\pm 0.017$)	0.927 ($\pm 0.042$)	0.103 ($\pm 0.066$)
0.60	Baseline	0.337 ($\pm 0.025$)	0.296 ($\pm 0.018$)	0.298 ($\pm 0.019$)	0.729 ($\pm 0.058$)	0.396 ($\pm 0.094$)
0.60	Proposed	0.279 ($\pm 0.016$)	0.731 ($\pm 0.043$)	0.397 ($\pm 0.022$)	0.803 ($\pm 0.049$)	0.259 ($\pm 0.081$)
0.50	Baseline	0.321 ($\pm 0.028$)	0.299 ($\pm 0.017$)	0.292 ($\pm 0.021$)	0.693 ($\pm 0.060$)	0.445 ($\pm 0.094$)
0.50	Proposed	0.267 ($\pm 0.014$)	0.609 ($\pm 0.040$)	0.355 ($\pm 0.019$)	0.619 ($\pm 0.060$)	0.503 ($\pm 0.099$)

The estimated graph $G(V,{\hat{E}})$ by the proposed method for one of the datasets with $p=0.95$ is shown in Fig. 3c. For the dataset, parameter $\theta$ is set to 287.2 from the estimated distribution (Fig. 3b). There are many falsely detected edges but all the edges in *E* are correctly detected and all the falsely detected edges keep the correct layer structure. The frequencies of the best grid values $\beta$ for the bandwidth of kernel density estimation are shown in Fig. 3d, which says that $\beta =0.2\sim 0.9$ are appropriate for these datasets.

### Application to real-world datasets

For real-world datasets, there is no ground truth graph so the measures adopted for synthetic datasets cannot be used to evaluate performance. Thus, only what we can do is to visually check the consistency of the estimated propagation graphs with given datasets. As for parameters of kernel density estimation, we use Gaussian kernel and set the bandwidth-related parameter $\beta$ to 0.25 because $\beta =0.2\sim 0.3$ are appropriate values for both the real-valued and binary state propagations in the experiments using synthetic datasets.

#### Stock price analysis

**Figure 4:**
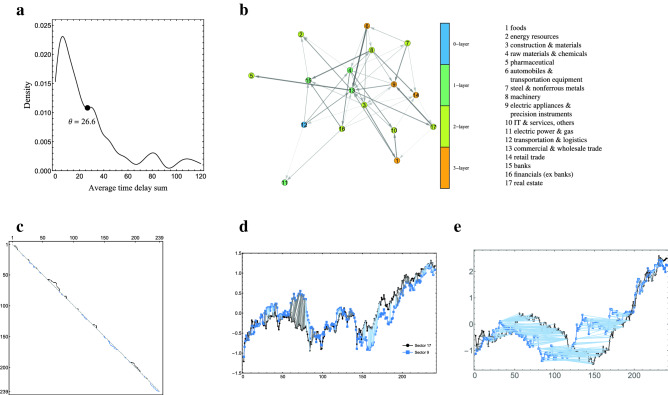
Results for stock market datasets. (**a**) Probability density of average time delay sum estimated by kernel density estimation for stock market datasets. We used Gaussian kernel with a bandwidth with the parameter $\beta = 0.25$. The black circle ($\theta = 26.6$) indicates a threshold value adopted by our method. (**b**) The estimated stock price propagation graph by the proposed method. Each vertex is labeled by its representing sector number. Each vertex’s color indicates its belonging layer: blue, green, yellow and orange vertices belong to $V^{{\hat{E}}}_0$, $V^{{\hat{E}}}_1$, $V^{{\hat{E}}}_2$, and $V^{{\hat{E}}}_3$, respectively. The thickness of an edge shows the size of the average time delay; the thicker the edge is, the longer the delay is. (**c**) The minimum cost alignment path between $s_{9}$ and $s_{17}$ in the dynamic programming table for the minimum cost alignment. The diagonal positions correspond to no delay, and above and below diagonal positions correspond to the statuses of delayed $s_9$ and $s_{17}$, respectively. (**d**) Line graph of time series $s'_{9}$ and $s'_{17}$ with their matched positions in the minimum cost alignment. The average time delay sum of $s_9$ from $s_{17}$ is 22.0. The horizontal axis is time, and the vertical axis is standardized stock price. Gray and light blue lines between $s'_{9}$ and $s'_{17}$ indicate estimated correspondences between the stock price derivative time series $s_{9}$ and $s_{17}$ in the minimum cost alignment. The gray (light blue) lines indicate that the sector 9 (sector 17) follows the sector 17 (sector 9). (**e**) The standardized sequences of the opening price for NAGAWA (black) and KYOKUTO BOEKI KAISHA (blue). Lines between them indicate their corresponding positions. The horizontal axis is time, and the vertical axis is standardized stock price. NAGAWA looks following KYOKUTO BOEKI KAISHA with large time delay during time period between 60 and 190.

We report our analysis of stock price propagation by the proposed method. Stock price fluctuates greatly and its propagation is ambiguous and the propagation direction often changes. In that sense, it does not seem to satisfy Assumption 1, but our method can be used to extract a trend such as which stock price tends to follow which stock price during the period in total. Here, we show the result of such trend analysis using opening stock price time series for one year period. We used the datasets of stock price time series of 2145 companies listed on the first section of the Tokyo Stock Exchange for the period from 4th January to 30th December in 2019. The set of the listed companies is partitioned into 17 sectors by TOPIX-17 series^31^. The given time series $p_j[t]$ ($t=0,\dots ,240$) is the sequence of the opening stock price of company *j* on *t*th day for $j=1,\dots ,2145$. We standardized each time series $p_j$ to $p'_j$ so that $p'_j[t]$ ($t=0,\dots ,240$) have mean zero and standard deviation one. The time series $s'_i[t]$ ($t=0,\dots ,240$) is the standardized sequence of the opening stock price on *t*th day averaged over companies in sector *i* for $i=1,\dots ,17$. Then, $s_i[t]$ ($t=1,\dots ,239$, $i=1,\dots ,17$), which is an estimated derivative of $s'_i$ at time *t*, is calculated by equation $s_i[t] = \frac{(s'_i[t]-s'_i[t-1]) + (s'_i[t+1]-s'_i[t-1])/2}{2}$. Figure 4b shows the estimated propagation graph with the vertices of 17 sectors by the proposed method for threshold $\theta = 26.6$, which is determined from estimated distribution of average time delay sum (Fig. 4a). Figure 4c shows the minimum cost path between the time series $s_{9}$ and $s_{17}$ in the dynamic programming table for calculating the minimum cost, which is composed of the optimally matched positions between the two time series. The horizontal and vertical axes are positions of $s_{9}$ and $s_{17}$, respectively, and the points above the diagonal line (black points) mean that $s_{9}$ is delayed from $s_{17}$ at those positions and the points below the diagonal line (light blue points) means that $s_{17}$ is delayed from $s_{9}$ at those positions. The average time delay sum of $s_{9}$ from $s_{17}$ is 22.0, which means that $s_{9}$ tends to follow $s_{17}$ in total. In fact, comparing to the diagonal line, there are more above points than the below points. Figure 4d shows the line graph of time series $s'_{9}$ and $s'_{17}$ with gray and light blue lines connecting their corresponding matched positions in the alignment. You can see that $s_9$ (derivative of $s'_9$) follows $s_{17}$ during two long time periods [59, 77] and [193, 208] with small time delays.

Among the set of pairs of individual stocks, stock pairs that have clearer leader-follower relationship can be found. Figure 4e shows the standardized sequences of the opening price for one of those pairs (“NAGAWA”, “KYOKUTO BOEKI KAISHA”) with the lines connecting corresponding points between them. In the figure, you can see that black stock (NAGAWA) follows blue stock (KYOKUTO BOEKI KAISHA) with large time delay during period between 60 and 190.

#### Cell’s firing analysis

We applied our method to estimating firing state propagation order of biological cells. The dataset is composed of 250-frame $\{0,1\}$-state and 2D-location sequences of 172 cells, where states 1 and 0 represent firing and not firing, respectively. Our method uses state sequences alone and location sequences are used only for result visualization.

We used the data of 144 cells except for 28 cells which could not be measured properly due to noise. From the set of 144 binary sequences with length 250, we extracted 4 datasets $I_1, I_2, I_3$ and $I_4$, each of which is composed of 144 length-100 consecutive subsequences starting at frame $t=1, 51,101$ and 151, respectively, of the original length-250 sequences.

**Figure 5:**
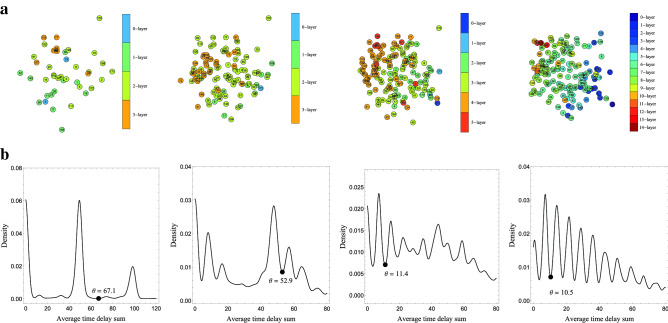
Estimated layer partitions and probability densities of the average time delay sum. (**a**) Layer partitions of the estimated propagation graphs for $I_1$ (cell location: $t=50$), $I_2$ (cell location: $t=100$), $I_3$ (cell location: $t=150$), $I_4$(cell location: $t=200$), respectively from left. (**b**) Probability density of average time delay sum estimated by kernel density estimation for each dataset; $I_1$, $I_2$, $I_3$, and $I_4$, respectively from left. We used Gaussian kernel with a bandwidth with the parameter $\beta = 0.25$. The black circles indicate threshold values adopted by our method.

**Figure 6:**
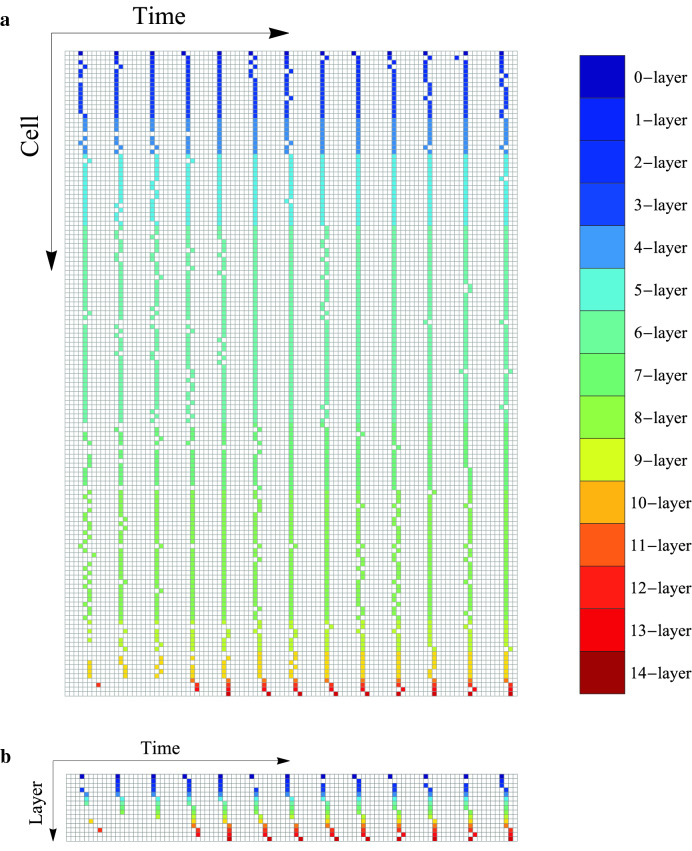
(**a**) $\{0,1\}$-cell-state sequences for period $I_4$. Each row represents the state sequence of the corresponding cell. Colored and blank states are firing (1) and non-firing (0) states, respectively, and color indicates each cell’s belonging layer. (**b**) Layer-consensus $\{0,1\}$-cell-state sequences for period $I_4$. The *i*th row represents the consensus state sequence among the *i*th layer cells, where the consensus state at time *t* means the majority state at that time.

The layer partitions of the estimated graphs by the proposed method for thresholds $\theta = 67.1(I_1), 52.9(I_2), 11.4(I_3), 10.5(I_4)$ are shown in Fig. 5a, where $\theta$s are determined from estimated distributions of average time delay sum (Fig. 5b). For datasets $I_2, I_3$ and $I_4$, the first layer’s cells look located around the lower right and the last layer’s cells look located around the upper left, and the locational direction of layer sequence $V^{{\hat{E}}}_0, V^{{\hat{E}}}_1,\cdots$ looks from the lower right to the upper left. Figure 6a shows $\{0,1\}$-state sequences in dataset $I_4$. We can see that cells with similar sequences are classified into the same layer. Appropriateness of the estimated layer order can be also confirmed by Layer-consensus state sequences shown in Fig. 6b.

## Concluding remarks

We proposed the way of constructing a state propagation graph that visualizes the estimated state propagation order of individuals. According to our experiments using real-valued and symbolic time series synthetic datasets generated by stochastic delay models, the edge sets of propagation graphs estimated by our method achieved comparable or higher F-measure and *layer accuracy* than those by a baseline method (optimal constant delay model), where layer accuracy is the accuracy on the number of steps to be taken in propagation from the source individuals to each individual.

In order to demonstrate practical usefulness of our method, we applied our method to propagation analyses of stock price and biological cell firing. For both datasets, the propagation order estimated by our proposed method is shown to be consistent with visually recognizable propagation order. The propagation delay is not stable for stock price propagation, but which stocks tended to follow which stocks in a given period is interesting information and automatic visualization may be useful to investors. Our method is considered to be useful for analyses of such unstable propagation.
